# Bioenergy‐Based Closed‐Loop Medical Systems for the Integration of Treatment, Monitoring, and Feedback

**DOI:** 10.1002/smsc.202300043

**Published:** 2023-08-22

**Authors:** Guanghui Zhang, Yandong Chen, Weixian Zhou, Chunying Chen, Ying Liu

**Affiliations:** ^1^ CAS Key Laboratory for Biomedical Effects of Nanomaterials and Nanosafety & CAS Center for Excellence in Nanoscience National Center for Nanoscience and Technology of China Beijing 100190 P. R. China; ^2^ University of Chinese Academy of Sciences Beijing 100049 P. R. China; ^3^ GBA National Institute for Nanotechnology Innovation Guangzhou 510700 Guangdong P. R. China

**Keywords:** bioenergy, closed-loop medical systems, feedback, self-powered, wearable healthcare systems

## Abstract

Wearable healthcare systems have captured the interest of researchers because they enable smarter and more personalized healthcare. However, these systems are limited by a separation between biosensors and therapeutic units that results in interrupted treatment and compromised patient recovery. It is therefore imperative to develop wearable closed‐loop medical devices that fully integrate physiological/pathological monitoring, signal feedback detection, diagnostics, and on‐demand therapeutic administration. Such systems require safe, sustainable, and continuously operating power sources, and bioenergy has gained attention in this regard because it can be sourced continuously from the human body without requiring substantial rigid energy storage space. The effective utilization of bioenergy would enable the realization of a self‐driven closed‐loop medical system with treatment‐monitoring feedback.

## Graphical abstract

Bioenergy is a green, accessible, and sustainable energy source. Personalized and intelligent medical treatment is more in line with people's needs. Closed‐loop medical systems integrate diagnostic, therapeutic, and feedback components. Bioenergy is the future power source for wearable medical devices.

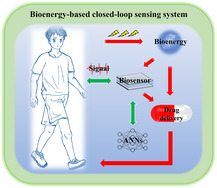

## 1 Introduction

Advances in wireless sensing and electronics technology have improved the miniaturization, integration, and intelligence of wearable devices.^[^ ^1^ ^]^ These systems hold extraordinary potential for biomedicine and the construction of feedback‐controlled closed‐loop systems that can perform diagnostics,^[^ ^2^ ^]^ real‐time monitoring,^[^ ^3^ ^]^ and on‐demand therapeutic delivery.^[^ ^4^ ^]^

Chronic and infectious diseases have always posed dangers to human health (e.g., recalcitrant wounds, tissue and organ inflammation, and COVID‐19)^[^ ^5^ ^]^ and are challenging to treat because such diseases can develop unpredictably and patient examination is often inconsistent. A diagnosis must be made before choosing and administering a treatment protocol or drug,^[^ ^6^ ^]^ but the traditional treatment process usually lacks timely dynamic monitoring of disease states. The result is poorly timed treatment interventions that reduce drug efficacy and introduce potential dangers from delayed treatment,^[^ ^7^ ^]^ and disruptive technologies are required to overcome these limitations. Some success has already been achieved with real‐time monitors of physiologic parameters (e.g., body temperature, heart rate, and blood sugar level),^[^ ^8^ ^]^ but the traditional energy supplies in these devices are not suited for advanced medical technology and personalized medicine. Recently, interest has developed in harvesting energy directly from the body to power medical equipment as studies indicate that bioenergy is a favorable renewable energy source for wearable medical devices.^[^ ^9^, ^10^ ^]^

The traditional power supply battery is a rigid device that requires replacement or recharging and therefore cannot satisfy the growing requirements for comfort, portability, and integration in wearable devices and medical equipment overall.^[^ ^11^ ^]^ This equipment relies on external near‐infrared light because energy is limited in time and space. The practice of medicine is gradually shifting away from healthcare facilities (e.g., hospitals and clinics) and toward time and location‐independent physiological monitoring.^[3c]^ Recent study has shown that wearable devices can detect COVID‐19 infection,^[^ ^12^ ^]^ and when combined with computer technology these smart wearables can discern authentic from false‐positive infections. These smart wearable devices are an ideal application for renewable bioenergy, including biomechanical, biochemical, and biothermal energy. Biomechanical energy can be scavenged from the soles of the feet, knees, ankles, wrists, the vibrations of organs, and other highly mobile areas.^[^ ^13^ ^]^ Biochemical energy can be harvested from sweat, tears, urine, and interstitial fluids,^[^ ^14^ ^]^ while biothermal energy can be obtained from temperature differentials between the body and the environment or between different body parts.^[^ ^15^ ^]^ This energy is largely wasted in daily life but might become useful for closed‐loop medical systems if wearable devices can harness these easily acquired and sustainable potential bioenergy sources. Closed‐loop medical systems will also require more advanced and intelligent sensing systems that, when integrated with renewable bioenergy sources, will enable intelligent biomedical devices with applications in eye diseases,^[4a]^ ear diseases,^[^ ^16^ ^]^ nonsuture wound diagnosis and treatment,^[^ ^17^ ^]^ and drug release. The application of bioenergy to closed‐loop treatment provides the basis for establishing personalized, intelligent, and integrated medical systems with broad development potential.

**Figure 1:**
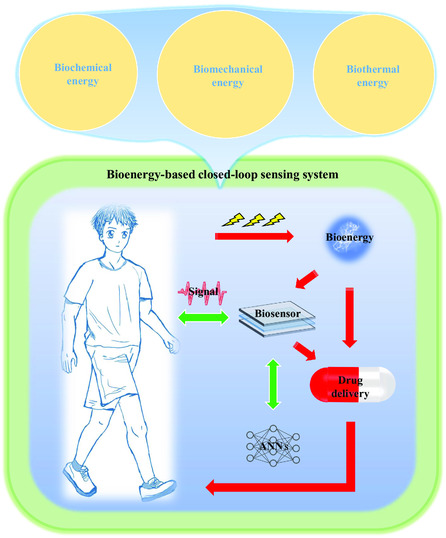
Schematic diagram of a bioenergy‐based closed‐loop medical system. The closed‐loop medical system can monitor the physiological signals of the human body in real time, realize on‐demand drug delivery through signal sensing, intelligently analyze the sensing signals through wireless sensing technology and machine learning (artificial neural networks, ANNs), and finally, feedback to the treatment system to achieve personalization and precision chemotherapy.

This comprehensive review presents a systematic survey of recent developments in closed‐loop medical systems for therapy, real‐time monitoring, and feedback regulation (see **Figure** **1**). We aim to provide a holistic understanding of the bioenergy‐based closed‐loop medical systems while identifying outstanding challenges and potential solutions and opportunities for technological advancement. Ultimately, our goal is to facilitate and expedite the clinical application of medical systems that integrate real‐time monitoring, therapeutic delivery, and feedback regulation.

## 2 Treatment‐Monitoring‐Feedback in a Closed‐Loop Medical System

Many current treatment measures are discrete and lack continuity, resulting in interrupted and limited patient recovery. Changing disease pathology is difficult to assess, often causing secondary damage upon reexamination while increasing medical supply and staffing costs.^[^ ^18^ ^]^ An established treatment‐monitoring feedback closed‐loop medical system would alleviate these problems.

### 2.1 Treatment Methods for Pathological Tissues

Treatment decisions must consider the therapeutic drug, the mode of administration, and the choice of drug carrier material; for a given disease there are typically multiple treatment options. For example, antibiotics can be used to kill bacteria in infected wound sites,^[^ ^19^ ^]^ but the synergistic combination of silver nanoparticles with antimicrobial peptides has an even greater antibacterial effect.^[^ ^20^ ^]^ Regarding drug delivery, the traditional methods of injection or oral administration are predominant, but transdermal and topical administration routes are of increasing interest because they avoid the first‐pass effects and discomfort of conventional methods.^[^ ^8^ ^]^ Transdermal and topical drug delivery approaches must consider many factors including species, molecular size and structure of the drug, temperature, and the pH of the drug delivery environment.^[^ ^21^ ^]^ For example, skin permeability is similar in humans and pigs but greater in mice.^[^ ^22^ ^]^

**Figure 2:**
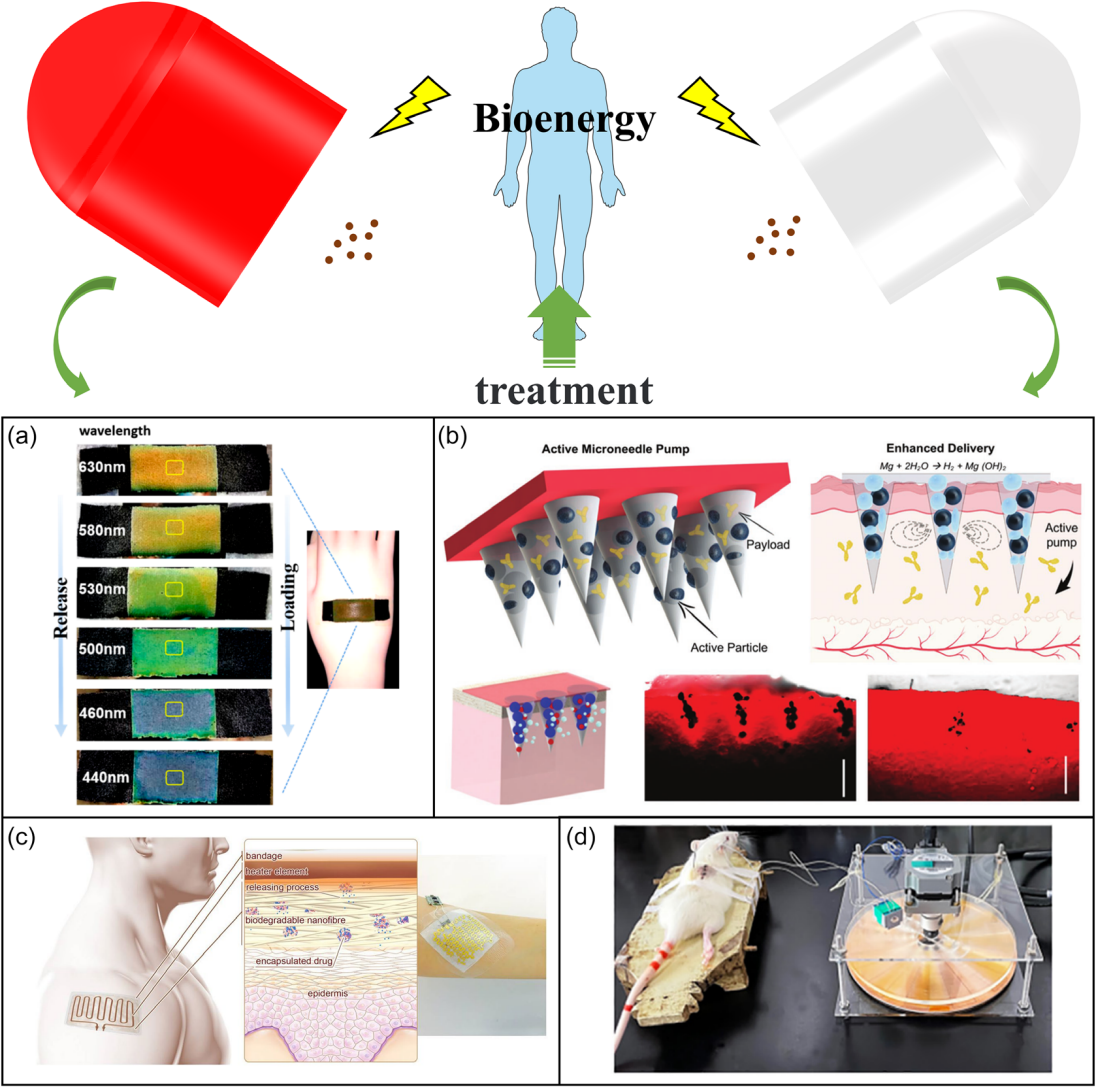
Therapeutic modalities can be applied in bioenergy‐based closed‐loop medical systems. a) Thermochromic color‐changing hydrogel functionalized textiles for drug release in vitro. Reproduced with permission. ^[^ ^31^ ^]^ Copyright 2020, American Chemical Society. b) Built‐in active microneedle patch with enhanced autonomous drug delivery. Reproduced with permission.^[^ ^38^ ^]^ Copyright 2019, Wiley‐VCH. c) Biodegradable elastic nanofibrous platforms with integrated, flexible heaters for on‐demand drug delivery. Reproduced under the terms of the CC‐BY Creative Commons Attribution 4.0 International license (https://creativecommons.org/licenses/by/4.0).^[^ ^19^ ^]^ Copyright 2017, The Authors, published by Springer Nature. d) Electroacupuncture stimulation promotes functional repair of spinal cord injury. Reproduced with permission.^[53b]^ Copyright 2022, Elsevier.

Hydrogels are widely used in transdermal and topical drug delivery and have recently trended toward multifunctionalized designs.^[^ ^1^, ^23^ ^]^ For example, nanomaterials endow a hydrogel with enhanced in vivo therapeutic functionality while preserving the original properties of the hydrogel, and such materials can be used in vivo in many diagnostic and therapeutic applications across several organs (e.g., skin wound healing, liver hemostasis, cardiac drug delivery, and bladder imaging via magnetic resonance).^[^ ^24^ ^]^ Conductive hydrogels that are responsive to matrix metalloproteinases can release basic fibroblast growth factor on demand to treat spinal cord injuries.^[^ ^25^ ^]^ Injection of agarose–gelatin–polypyrrole composite hydrogels in vivo can completely cover tissue defects and provide a biocompatible microenvironment for neural migration and stem cell differentiation.^[^ ^26^ ^]^ Ferromagnetic fluid hydrogels prepared with iron tetrasulfide (Fe_3_S_4_), carboxymethyl chitosan, and gold can release hydrogen sulfide in acidic environments to inhibit neuroinflammation.^[^ ^27^ ^]^ Injectable hydrogels can also treat closed and difficult‐to‐close wounds.^[^ ^28^ ^]^ A double‐barreled syringe allows in vivo mixing of hydrogel monomers to achieve in situ gelation and precise wound site treatment via loaded drug nanoparticles.^[^ ^24^, ^29^ ^]^ Bioenergy‐driven hydrogels can be used more intelligently for therapy. The injectability and responsiveness of thermal and pH‐sensitive hydrogels facilitate drug loading and delivery in situ. Hybrid hydrogel–fabric dressings can compensate for the poor mechanical properties of pure hydrogel dressings. UV‐responsive antimicrobial hydrogels have been used in wound dressings.^[^ ^30^ ^]^ A hydrogel‐functionalized textile loaded with poly(*N*‐isopropylacrylamide*‐co*‐acrylic acid) can release drugs on demand through mild thermal stimulation (**Figure** **2**a).^[^ ^31^ ^]^ Poly(3,4‐ethylenedioxythiophene) (PEDOT) is a promising material with excellent biocompatibility and electrical properties and has many applications in drug transport. Alginate hydrogels loaded with heat‐sensitive drugs can be fabricated into a smart, flexible bandage.^[^ ^32^ ^]^ A self‐regulating drug release system uses dexamethasone–PEDOT conjugates linked by biochemically labile bonds,^[^ ^33^ ^]^ and PEDOT:polystyrenesulfonate (PSS) hydrogels and hydrogel fibers can be used to develop soft and self‐healing bioelectronic devices.^[^ ^34^ ^]^ Conductive hybrid polymers of PEDOT:poly(dimethylacrylamide*‐co*‐4‐methacryloyloxy benzophenone (5%)‐*co*‐4‐styrene sulfonate (2.5%)) conductive polymers can deliver drugs when electrically stimulated and have a greater drug storage capacity than traditional PEDOT:PSS systems.^[^ ^35^ ^]^

Microneedle‐mediated transdermal drug delivery is a convenient method for patients with chronic disease (e.g., hypertension, diabetes, and Alzheimer's) because the drug is painlessly self‐administered and generates no biohazardous waste.^[4c]^ Microneedles can be made of metallic or nonmetallic materials tailored to treat many diseases and effervescent microneedles can be designed for diseases that require continuous and chronic treatment.^[^ ^36^, ^37^ ^]^ Upon penetrating the skin, the microneedle tip reacts with skin tissue fluid (Figure 2b) and separates from the patch for long‐term drug release.^[^ ^38^, ^39^ ^]^ Microneedles can also combine with longer‐lasting contact lenses to treat eye conditions such as glaucoma.^[^ ^40^ ^]^ Mesoporous microneedles can painlessly penetrate the stratum corneum to deliver material subcutaneously, and drug delivery can be enhanced when combined with iontophoresis.^[^ ^41^ ^]^ Microneedles can deliver drugs orally to the intestines and stomach,^[^ ^42^ ^]^ thereby serving as an oral delivery platform for high‐molecular‐weight drugs that avoids needle waste and harm to the gastrointestinal tract.^[4d]^ Microneedle patches can also perform vaccine inoculation against Ebola virus^[^ ^43^ ^]^ and COVID‐19^[^ ^5^ ^]^ with reduced training requirements for vaccinators. Implantable microneedles can extend the delivery of loaded antigens to provide more sustained immune stimulation and improved immunogenicity. For example, earlier use of a subcutaneously implanted chitosan array‐like microneedle patch loaded with ovalbumin as a model antigen has demonstrated an ovalbumin‐specific immune response lasting at least 6 weeks in rats.^[^ ^44^ ^]^ Moreover, the microneedles were integrated with a triboelectric nanogenerator (TENG) that converts the mechanical energy of finger slides into electrical energy that powers a transdermal electrical stimulation system for facilitating drug penetration.^[^ ^45^ ^]^

The combination of hydrogels with microneedles and ultrasonic^[^ ^46^ ^]^ drug delivery creates a viable alternative to traditional injection methods.^[^ ^47^ ^]^ A double‐layered adhesive microneedle patch based on mussel adhesion protein is effective for tissue delivery in vitro and in vivo.^[^ ^48^ ^]^ Hydrogel–microneedle hybrids can deliver temperature‐responsive drug formulations^[^ ^49^ ^]^ (e.g., antibiotics^[^ ^50^ ^]^ and growth factors) with on‐demand drug release.

In addition to hydrogels and microneedles, electrospun fibers are suitable for wounds. Polyacrylamide hydrogels can be synthesized with amoxicillin‐loaded polyaniline nanofibers of a large aspect ratio to allow precision drug release under electrical stimulation.^[^ ^51^ ^]^ Electrospun fibrous scaffolds are easily prepared and highly versatile materials with surface chemical properties that favor drug adsorption,^[^ ^52^ ^]^ and the high porosity and large specific surface area of the scaffold improve drug loading efficiency and delivery.^[^ ^50^ ^]^ Variants of the electrospinning technology include coaxial electrospinning, multiaxial electrospinning, and electrospraying. Electrospun drug stents enable controlled drug release to promote the regeneration of skin, nerve, heart, and other soft tissues and also serve as cancer treatments (Figure 2c).[^4^, ^19^]

Electroacupuncture is a unique electrical stimulation therapy in which a TENG inserted into the acupuncture points of mice can activate cells and promote the regeneration of nerve tissue (Figure 2d).^[^ ^53^ ^]^ Neural microelectrodes can release dexamethasone to treat local inflammation.^[^ ^54^ ^]^ Electrical stimulation promotes the regeneration and repair of bone and other tissues, thus highlighting the potential for physiologic regulation in biomaterial design.^[^ ^55^ ^]^ An electrogenic dressing combines TENG with negative‐pressure wound therapy to produce a stable, safe, high‐intrinsic electric field that promotes tissue remodeling and reduces scar formation.^[^ ^56^ ^]^ Status epilepticus, a fatal epileptic condition that requires immediate treatment, can be treated with a soft subcutaneous implantable drug delivery device.^[^ ^57^ ^]^ Atrial fibrillation can be treated with low‐level vagus nerve stimulation from a self‐powered closed‐loop bioelectrical device that monitors the patient's real‐time pulse wave status and stimulates corrective impulses automatically during the development of atrial fibrillation.^[^ ^58^ ^]^ Taken together, these examples demonstrate the broad potential of biological energy for personalized medicine.

### 2.2 Wearable Devices for Real‐Time Monitoring

Real‐time physiologic monitoring of disease pathology enables more accurate treatment and repair.^[^ ^59^ ^]^ Sensors currently in clinical use include blood pressure oximeters, blood glucose meters, electrocardiographs, gastroenteroscopes, and infrared radiation thermometers,^[^ ^60^ ^]^ but these bulky instruments are difficult to transport and store at medical institutions. In contrast, wearable medical devices are small and portable with more comprehensive signal sources, thereby attracting attention for point‐of‐care testing.

Some smart wearable devices have already been well developed, and the accelerated commercial application of these devices (smartwatches and activity trackers) creates opportunities to apply artificial intelligence tools in support of digital healthcare.^[^ ^1^ ^]^ Previous studies have found that consumer devices can monitor the progression of respiratory and influenza‐like illnesses by collecting physiologic and behavioral data (e.g., resting heart rate, step count, sleep duration, and respiratory rate) along with patient‐reported symptoms.^[^ ^1^, ^61^ ^]^ However, the energy supplies and wearable comfort of these smart devices can be greatly improved.

**Figure 3:**
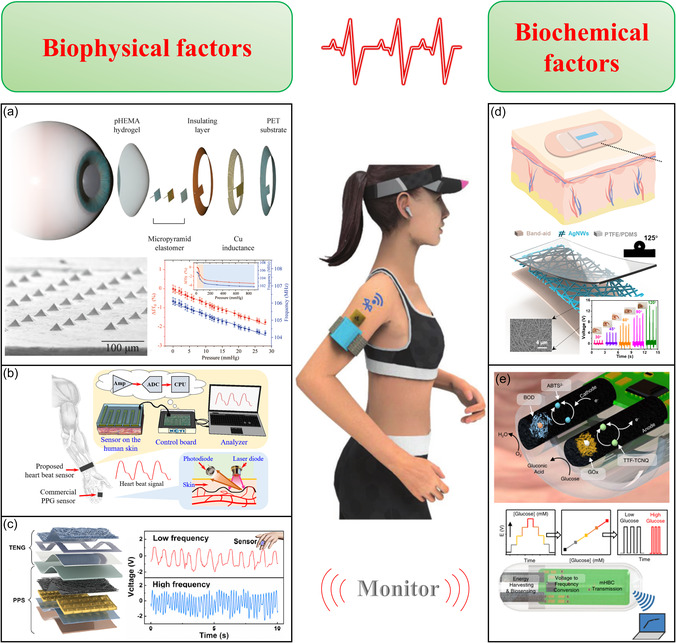
Self‐powered wearable device for real‐time monitoring of human biophysical and biochemical signals. Reproduced with permission.^[^ ^92^ ^]^ Copyright 2022, Wiley‐VCH. a) High‐sensitivity smart hydrogel contact lenses for wireless intraocular pressure monitoring. Reproduced with permission.^[^ ^77^ ^]^ Copyright 2022, American Chemical Society. b) Real‐time heartbeat wireless sensing. Reproduced with permission.^[^ ^89^ ^]^ Copyright 2017, Elsevier. c) High precision, real‐time sensing system for pressure‐sensitive electronic skin. Reproduced with permission.^[^ ^90^ ^]^ Copyright 2022, Elsevier. d) Self‐powered intelligent Band‐Aids for motion monitoring. Reproduced with permission. ^[^ ^98^ ^]^ Copyright 2022, Elsevier. e) Edible self‐powered wireless biosensing system for real‐time monitoring of gastrointestinal metabolites. Reproduced under the terms of the CC‐BY Creative Commons Attribution 4.0 International license (https://creativecommons.org/licenses/by/4.0).^[^ ^7^ ^]^ Copyright 2022, The Authors, published by Springer Nature.

To accommodate human movement while preserving comfort, wearable devices must be flexible and stretchable.^[^ ^62^ ^]^ Inkjet printing,^[^ ^63^ ^]^ screen printing, electrochemical deposition, 3D printing, electrospinning,^[^ ^64^ ^]^ plasma etching, and other methods can be used to fabricate wearable devices.^[^ ^65^ ^]^ The stretchable conductive parts are usually prepared by patterning metal film into a serpentine or wavy shape,^[11c]^ and conductive fillers (e.g., PEDOT:PSS, carbon nanotubes, graphene, and metal particles)^[^ ^63^, ^66^, ^67^, ^68^ ^]^ are used to enhance flexibility. In addition, poly(dimethylsiloxane) (PDMS),^[^ ^69^ ^]^ poly(ethylene glycol), poly(vinyl alcohol), polyurethane, and other elastomers have been doped in to form stretchable conductive composites.^[62b]^ For example, carbon aerogels can enhance PDMS nanocomposites for wearable multifunctional heating and sensing devices.^[^ ^70^ ^]^ A highly robust and self‐powered electronic skin with excellent mechanical toughness and self‐healing ability can be developed from conductive polyurethane elastomers with homogenous structure and friction.^[^ ^71^ ^]^ Hydrogels, especially self‐healing hydrogels,^[^ ^71^, ^72^ ^]^ are outstanding stretchable materials^[^ ^73^ ^]^ with similarities to human skin^[^ ^74^ ^]^ and combine well with other materials.^[^ ^75^ ^]^ Hydrogel systems can respond to various stimuli with excellent sensitivity, flexibility, and stability and have been used for wearable monitoring devices.^[^ ^76^, ^77^ ^]^ A poly(2‐hydroxyethylmethacrylate) hydrogel can meet the clinical requirements of intraocular pressure detection with a sensitivity of 1.101‰ mmHg^−1^ (**Figure** **3**a).^[^ ^77^ ^]^

Wearable devices can be integrated into fabrics to form excellent designs in which the stainless steel materials are wrapped in yarns to form conductive pathways,^[^ ^78^ ^]^ thus laying the foundation for fully flexible electronic textiles.^[^ ^79^ ^]^ These electronic textiles designs must also consider flame retardancy, antibacterial properties, oxidation resistance, and air permeability.^[^ ^80^ ^]^ Wearable devices can also be fabricated from MXene‐based multifunctional smart fibers with excellent electrical conductivity and mechanical strength. Supercapacitors based on this material have high energy density and working stability^[^ ^81^ ^]^ that, when integrated within flexible smart textiles, can maintain structural integrity and monitor deformation, friction, and water exposure. Sodium alginate fabrics functionalized with graphene oxide and polypyrrole can be prepared by hot press reduction and in situ polymerization, and these materials show promise as ultrasensitive NH_3_ gas sensors and wearable heaters for thermoresponsive drug delivery in renal diagnosis and point‐of‐care applications.^[^ ^79^ ^]^ Hydrogel‐functionalized textiles can be used for both in vitro drug release and synchronous visual monitoring.^[^ ^31^ ^]^

Wearable physical sensors have initially been used to monitor mobility and vital signs[^3^, ^82^] such as steps,[^3^, ^83^] calories burned, heart rate,^[^ ^84^ ^]^ blood pressure,^[^ ^85^ ^]^ breathing rate, skin temperature, and brain activity.^[^ ^86^ ^]^ Wearable devices play a key role in healthcare by providing a feedback system to monitor and evaluate a person's real‐time state.^[^ ^87^ ^]^ Angle sensors are critical detectors in robot‐assisted joint rehabilitation training that monitor the real‐time rotation direction and angle of a personal brace.^[^ ^88^ ^]^ A unit made of flexible piezoelectric polymers can monitor respiration, the carotid artery, and heartbeat (Figure 3b).^[^ ^89^ ^]^ A flexible piezoresistive pressure sensor integrated with a TENG can detect fingertip pulse and finger tremors with great sensitivity and can distinguish different walking postures (Figure 3c).^[^ ^90^ ^]^ An electronic skin (MPM E‐Skin) based on MXene–polyurethane mesh has been reported to have very low electrode‐to‐skin contact impedance, a high signal‐to‐noise ratio, high air permeability, an extensive strain measurement range, and unique segmental sensitivity. This electronic skin is suitable for detecting physiological signals such as pulse, sound, and joint movement.^[^ ^91^ ^]^ A self‐powered wearable sweat analysis system was designed to enable the wireless monitoring of concentration fluctuations of Na^+^ and K^+^ in sweat.^[^ ^92^ ^]^ Wearable stress‐monitoring devices can extract real‐time vital signs such as heart rate, respiratory rate, and blood pressure to detect patient status regarding venous thromboembolism, arrhythmia, seizure detection, stress assessment, and sleep.[^64^, ^93^] Nanomembrane electrode arrays enable electromyography recording from a large surface area on the skin and can be used for operant modulation of the Hoffmann reflex in patients with dyskinesia after spinal cord injury.^[^ ^94^ ^]^ A wearable device can assess subjective emotional value by recording facial muscle electromyography of the corrugator supercilii and zygomaticus major.^[^ ^95^ ^]^ Wearable temperature sensors^[^ ^12^ ^]^ can collect continuous body temperature data to detect the high fever symptoms of COVID‐19 infection and onset.^[^ ^96^ ^]^ Respiratory infections can be diagnosed by monitoring changes in the nocturnal longitudinal respiratory rate.^[^ ^97^ ^]^ Graphene film‐based pressure sensors are ideal for monitoring pulse, respiration, and motion.^[^ ^86^ ^]^ A traditional bandaid can be coated with a silver nanowire network and polytetrafluoroethylene/PDMS mixture to provide cheap, convenient, and effective multifunctional human health sensing and monitoring (Figure 3d).^[^ ^98^ ^]^ To achieve more highly integrated portable smart electronics that can transmit Morse and Gray codes for text messages or remote control of electronic devices, TENGs have also been used to fabricate self‐powered hybrid encoders.^[^ ^99^ ^]^

Wearable biosensors can inform disease diagnostics by monitoring clinically relevant biomarkers via chemical reactivity (Figure 3e).^[^ ^7^, ^17^, ^100^ ^]^ Multifunctional microgel polymers can monitor multiple health signals because the binding of physiologically relevant metabolites (e.g., uric acid and bacterial metabolites in sweat) promotes microgel polymer conformational changes that alter capacitance.^[^ ^101^ ^]^ Advances in biological sample collection and processing, microfluidic devices, and smartphone‐based data analysis have enabled wearable biosensors to continuously monitor biological fluids for physiological information. The widespread acceptance of wearable biosensors in clinical diagnostics requires a deep understanding of the relationship between biomarkers in blood and noninvasive physiological fluids.^[^ ^2^, ^3^, ^102^ ^]^ Because disease‐associated biomarkers are often present at substantially lower concentrations in noninvasive physiological fluids (10‐fold lower than in serum samples), noninvasive wearable sensors require high sensitivity and selectivity.^[^ ^103^ ^]^ Sensors integrated into gloves can selectively and noninvasively detect therapeutic drugs and biomarkers in sweat samples. This approach has shown promising results in detecting uric acid, paracetamol, paroxetine, and ethinylestradiol with high accuracy,^[^ ^104^ ^]^ and wearable devices can also detect inflammation and infection via the inflammatory cytokines in sweat.^[^ ^105^ ^]^

A pH‐sensitive colorimetric wound dressing enables real‐time monitoring for bacterial infections,^[^ ^106^ ^]^ and a potentiometric pH sensor can more accurately detect wound pH.^[62b]^ Temperature is also an important parameter and smart bandages that integrate temperature and pH sensors together have been developed.^[3a]^ Sweat metabolites such as glucose, lactic acid, urea, and electrolytes can be detected to assess health conditions,^[^ ^107^ ^]^ and sweat collection is a critical step in such measurements. Traditionally, the whole‐body flushing method is used, though in wearable devices a microfluidic‐embedded patch can collect sweat for analysis.^[^ ^108^ ^]^

Physiological signals can also be monitored from tears, urine, and wounds. At a wound site, synchronous in situ monitoring can detect infection via the principle of microbial response.^[^ ^109^ ^]^ Currently, these sensors detect the enzymatic reaction of lactate oxidase with lactic acid in sweat^[^ ^110^ ^]^ and use skin contact to improve sensitivity. A tattoo‐based electrochemical sensor can extract biological fluids via reverse ion import.^[^ ^14^ ^]^ A device using microfluidic storage can minimize direct skin contact and sample evaporation in glucose and lactic acid sensors,^[^ ^111^ ^]^ with the microfluidic channels providing rapid sampling and efficient analyte transport through the sensing electrodes. Wearable microfluidic device design must also consider stretchability and ductility.^[^ ^112^ ^]^ A triboelectric microfluidic device generates a voltage in response to the flow of target analyte through the microchannel,^[^ ^113^ ^]^ thus combining sensor technologies with microfluidics to analyze disease states and toxic materials. It is exhilarating to realize the miniaturization, integration, and automation of multiple detection methods on a single chip.

### 2.3 Wireless Sensing and Machine Learning‐Based Detection and Feedback

**Figure 4:**
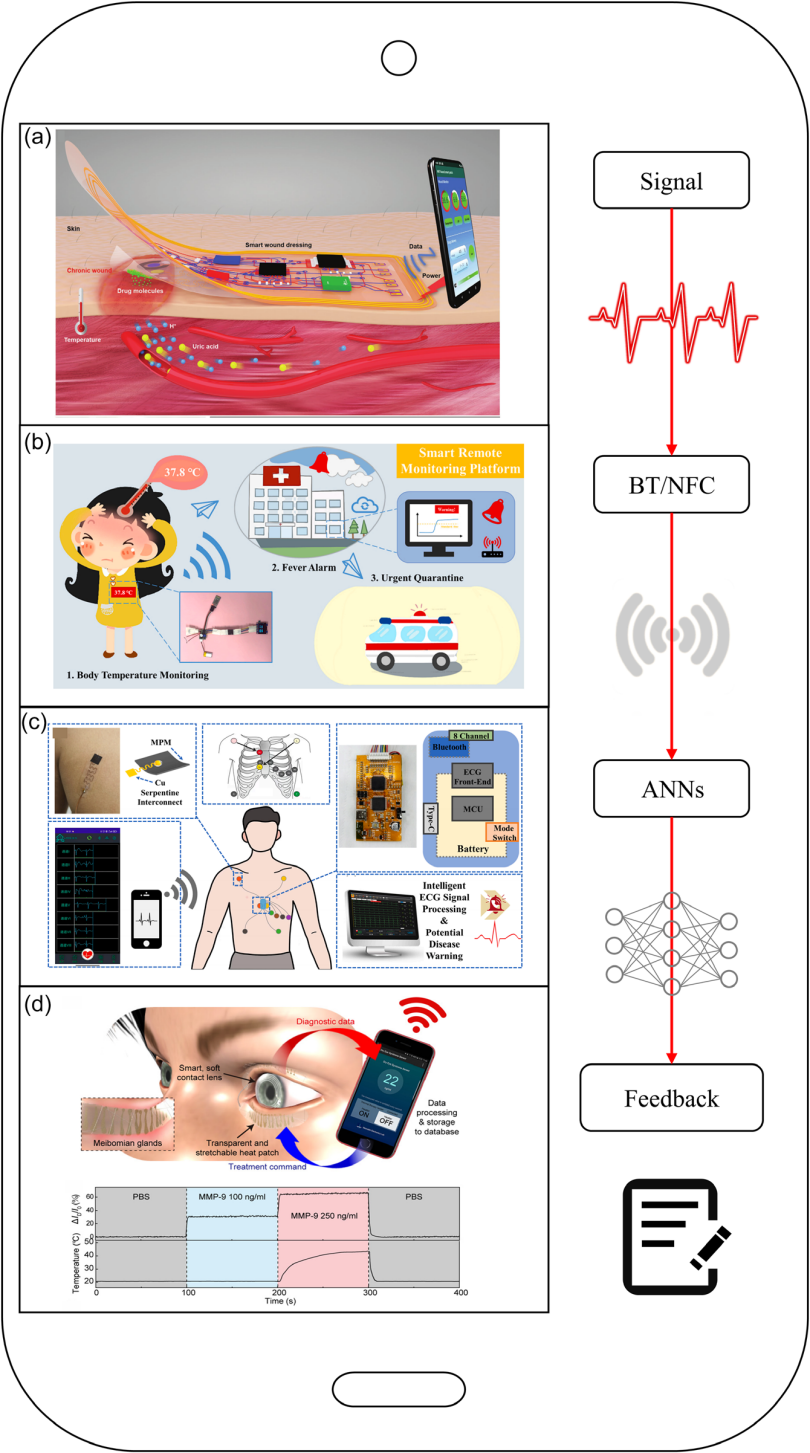
Wireless technology and machine learning enable the sensing and analytics feedback loop. a) Battery‐free and wireless smart wound dressing for wound infection monitoring and electrically controlled on‐demand drug delivery. Reproduced with permission.^[^ ^116^ ^]^ Copyright 2021, Wiley‐VCH. b) Real‐time temperature monitoring feedback. Reproduced with permission. ^[67a]^ Copyright 2022, Elsevier. c) Electronic skin for intelligent ECG monitoring. Reproduced with permission.^[^ ^91^ ^]^ Copyright 2023, Elsevier. d) Smart contact lenses and transparent heat stickers are used for remote monitoring of mobile phones. Reproduced with permission.^[^ ^57^ ^]^ Copyright 2021, The Authors, published by AAAS.

For traditional sensors, more extensive integration requires more complex circuits, and wireless sensor technologies (e.g., Bluetooth and near‐field communication) can address these issues.^[^ ^114^ ^]^ Bluetooth is a short‐range (10 m or less), low‐power, midfield communication standard that supports one‐to‐one or one‐to‐many communication.^[^ ^115^ ^]^ Near‐field communication is a short‐range, high‐frequency communication technology for contact‐free point‐to‐point data exchange and transmission between electronic devices (**Figure** **4**a).^[^ ^116^ ^]^ Smart wound dressings have already integrated near‐field communication to achieve on‐site signal processing and drug delivery control,^[^ ^117^ ^]^ and similar capabilities have been reported with Bluetooth integration. The database of collected signals is analyzed and the results are used for feedback regulation which can be applied to treat diseases of the nervous system; one study used statistical feature extraction and deep neural network classifiers to detect seizures.^[^ ^6^ ^]^ Wound infection has also been monitored to regulate on‐demand drug release and avoid side effects.^[^ ^118^ ^]^ Wireless sensing devices can be integrated with hydrogels, fabrics, and electrospun fibers;^[^ ^114^ ^]^ a hybrid silk yarn‐polyaniline‐carbon nanotube (SPC) device can be manually sewn into smart fabric and combined with a microcontroller, battery, Bluetooth module, buzzer, and LED lights to form a multifunctional wearable that monitors walking speed, push‐ups, and sitting posture.^[67a]^ More extensive component integration on a low‐power board can efficiently manage harvested bioenergy and minimize energy loss.^[^ ^92^ ^]^ An SPC with excellent temperature sensitivity can be assembled into intelligent clothing to monitor and wirelessly transmit body temperature data (Figure 4b).^[67a]^ An intelligent theranostic compression device with real‐time vital sign monitoring and automatic adjustment of compression levels can effectively prevent venous thromboembolism.^[^ ^119^ ^]^

The MXene–polyurethane mesh electronic skin system is integrated with a high‐performance convolutional neural network and long‐/short‐term memory paired with an intelligent electrocardiogram (ECG) algorithm. The ECG algorithm analyzes real‐time data during daily activities and provides diagnostics and corresponding health advice and early warnings (Figure 4c).^[^ ^91^ ^]^ The eye is a complex organ with many critical physiologic parameters (e.g., intraocular pressure, corneal temperature, and pH) and biomarker metabolites (e.g., glucose, proteins, and specific ions) worth monitoring.^[4a]^ Smart Contact Lenses with Bluetooth functionality can monitor the physiologic state of the eye and handle feedback signals to detect biomarkers of chronic superficial inflammation in tears and transmit diagnostics to a smartphone (Figure 4d).[^4^, ^57^] A data processing module can handle physiological information and activates a neural stimulator module; when stimulating electrodes are implanted in the medial forebrain bundle of running mice, a dramatic enhancement in endurance performance can be demonstrated.^[^ ^120^ ^]^ Heart rate‐enabled wearables can be used to measure respiratory rate; changes in night‐time longitudinal respiratory rate indicate respiratory infections such as COVID‐19.^[^ ^97^ ^]^ Self‐powered wireless transmission is critical for the development of wearable devices, while deeper physiologic signal processing will improve disease assessment and corresponding treatment decisions.

## 3 Bioenergy for Self‐Powered Devices

Energy sources for smart electronic devices and next‐generation wearable medical devices are critical concerns. Potential energy devices such as supercapacitors,^[^ ^121^ ^]^ fuel cells, solar cells, and nanogenerators can be used for personalized medicine and smart devices. Bioenergy refers to energy derived from the chemical, mechanical, and thermal energy existing on or within living organisms; these energy sources are converted to electrical energy that powers medical devices and improves their component integration.

### 3.1 Biochemical Energy

**Figure 5:**
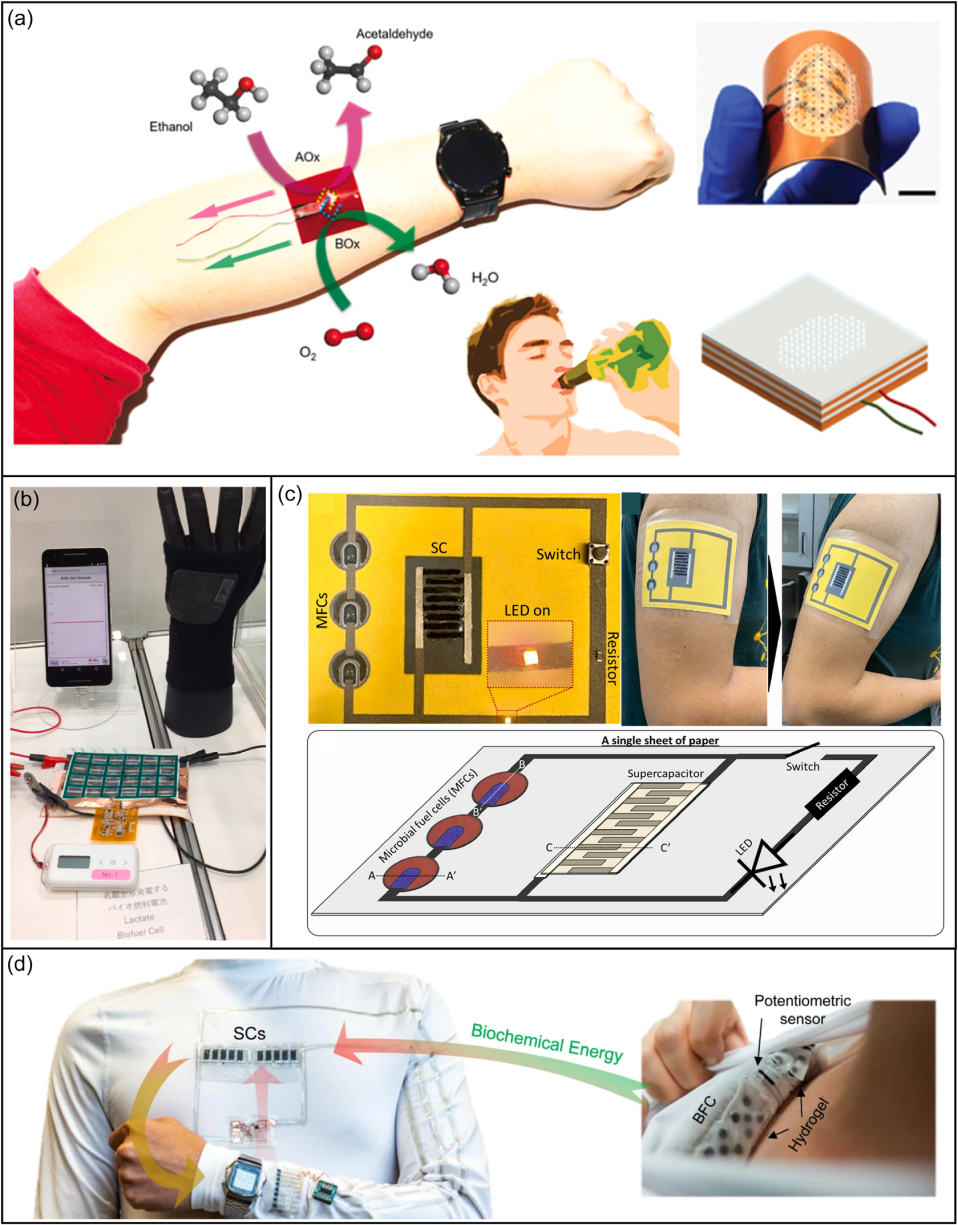
Biochemical energy in the closed‐loop medical system. a) Flexible wearable skin ethanol BFCs. Reproduced with permission.^[^ ^107^ ^]^ Copyright 2021, Elsevier. b) Paper‐based lactic acid BFC array. Reproduced with permission.^[^ ^131^ ^]^ Copyright 2021, Elsevier. c) Paper‐based self‐powered system for bio‐energy harvesting. Reproduced with permission.^[^ ^132^ ^]^ Copyright 2022, Elsevier. d) Wearable multimodule electronic textile bioenergy microgrid system. Reproduced under the terms of the CC‐BY Creative Commons Attribution 4.0 International license (https://creativecommons.org/licenses/by/4.0).^[^ ^133^ ^]^ Copyright 2021, The Authors, published by Springer Nature.

Biofuel cells (BFCs) are commonly used to harvest bioenergy via enzymatic reactions^[^ ^14^ ^]^ that utilize the remarkable specificity of enzyme‐substrate interactions and generate reaction products in proportion to the substrate concentration.^[^ ^110^ ^]^ Unlike traditional rigid energy storage systems (rechargeable batteries and supercapacitors),^[^ ^122^ ^]^ wearable BFCs generate green electricity from energy‐dense, carbon‐neutral fuels via efficient bioelectrochemical reactions; lactate and glucose are two of the most common substrates. These cells have excellent biocompatibility, remarkable environmental sustainability, and outstanding miniaturization potential.^[^ ^123^ ^]^ BFCs are classified by electron transfer pathways into mediated electron transfer cells and direct electron transfer cells.^[^ ^14^ ^]^ In practice, BFCs can noninvasively harvest biochemical energy from biological fluids such as sweat, saliva, interstitial fluid, and tears;^[^ ^124^ ^]^ the fluid provides the electrolyte (e.g., glucose,^[^ ^125^ ^]^ lactic acid,^[^ ^126^ ^]^ and ethanol)^[^ ^107^, ^127^ ^]^ that fuels power generation (**Figure** **5**a).^[^ ^108^ ^]^

A BFC that harvests chemical energy from the finger sweat of a 2 cm^2^ area can collect hundreds of millijoules of energy during sleep.^[^ ^126^ ^]^ When combined with traditional textile technology, these cells enable wearable bioenergy harvesting technology driven by human sweat.^[^ ^128^ ^]^ Functional yarns can be woven to extend horizontally structured microbial fuel cells into 2D and 3D wearable textiles.^[^ ^129^ ^]^ A BFC bracelet can harvest 2.0 V (sufficient to power a digital wristwatch) from human sweat using lactate oxidase/ozone mediator/carbon nanotube fibers to process lactic acid. The layered cell generates 74 μW at 0.39 V in 20 mM artificial sweat lactate and maintains performance at over 80% for 12 h.^[^ ^130^ ^]^ A paper‐based BFC generates a maximum open‐circuit voltage of 3.66 V and a current of 1.8 mA from the lactic acid in human sweat. The cell generates 4.3 mW which can power various miniature electronics and wireless devices (Figure 5b).^[^ ^131^ ^]^ A needle‐like BFC composed of enzyme/mediator/carbon nanotube composite fibers generates electricity from glucose when inserted into fruit or animal bodies.^[^ ^125^ ^]^ Battery output power could be improved by integrating multiple devices. Six‐layer BFCs integrated into bandages and sportswear can generate enough electricity from sweat to directly power sports watches.^[128b]^ A paper integrated with three microbial fuel cells as energy harvesters and a solid‐state supercapacitor as an energy storage device can generate an output of 4 μW cm^−2^ and 37 μA cm^−2^ from sweat. The device stores 9.81 mF of energy and exhibits stable capacitive behavior over 100 cycles with excellent self‐charging properties (Figure 5c).^[^ ^132^ ^]^ Biochemical and biomechanical energy is collected using perspiration‐based BFCs and triboelectric generators, respectively. The energy collected by supercapacitors generates high power output in a multimodule bioenergy microgrid system (Figure 5d).^[^ ^133^ ^]^

Because biofluid‐based fuel cells use fuel sources that also contain important physiologic metabolites (e.g., sweat). They may serve simultaneously as biosensors.^[^ ^8^, ^111^ ^]^ A battery‐free sweat‐sensing system that integrates antisweat functionality, self‐sustaining energy, and a wireless communication interface can transmit relevant measurements from sweat (including [Na^+^], [K^+^], and pH) to a user interface.^[^ ^134^ ^]^ Additionally, closed‐loop drug release may be controlled by feedback from continuous metabolite monitoring. Drugs can be loaded into the electrolyte or electrodes of the wearable BFC for on‐demand release, with the target metabolite serving as both analyte and fuel. Wearable BFCs can also be incorporated within smart wound dressing in which the current generated by the fuel cell promotes wound healing and drug release (e.g., anti‐inflammatory drugs and antibiotics).^[^ ^110^ ^]^ A self‐powered drug release system has been fabricated based on a redox polymer‐mediated glucose BFC in which a drug is released dose dependently in response to the power density of a stimulating current.

A glucose/O_2_ BFC‐based antibiotic delivery system for ampicillin was developed using a similar strategy.^[^ ^135^ ^]^ An organic iontophoresis patch with integrated BFC, fuel (fructose), and drug‐releasing hydrogel electrolytes formed a complete wearable BFC^[129a]^ in which dermal penetration of ascorbic glucoside and rhodamine‐B was assisted by current stimulation. Microneedles can also be combined with wearable BFCs to deliver macromolecular drugs.^[^ ^110^ ^]^ An edible biosensing system with self‐powered glucose BFCs has also monitored metabolites in the small intestine.^[^ ^7^ ^]^

Wearable BFCs have outstanding biocompatibility, miniaturization potential, and multifunctionality, but these devices still face several key challenges that must be addressed: 1) low cell voltage, 2) low power output, and 3) short biocatalyst lifetime.

### 3.2 Biomechanical Energy

**Figure 6:**
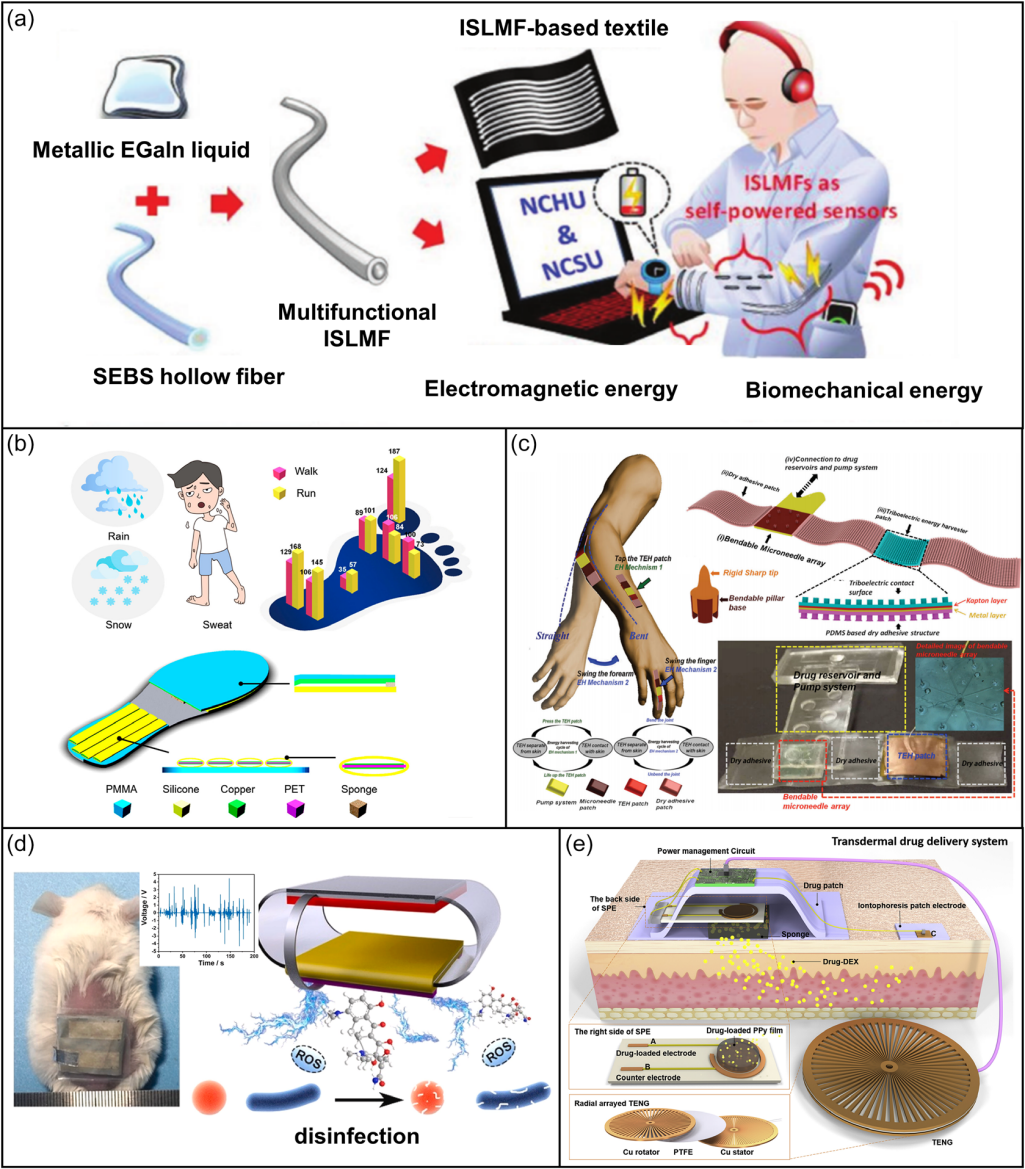
Biomechanical energy in the medical system. a) Elastic multifunctional liquid metal fibers for collecting mechanical energy. Reproduced with permission.^[^ ^136^ ^]^ Copyright 2021, Wiley‐VCH. b) Insoles for harvesting biomechanical energy. Reproduced with permission.^[140c]^ Copyright 2020, American Chemical Society. c) Self‐powered bendable microneedle array patch for transdermal drug delivery. Reproduced under the terms of the CC‐BY Creative Commons Attribution 4.0 International license (https://creativecommons.org/licenses/by/4.0).^[152b]^ Copyright 2016, The Authors, published by Wiley‐VCH. d) TENG for promoting healing of infected wounds. Reproduced with permission.^[^ ^155^ ^]^ Copyright 2021, Elsevier. e) On‐demand transdermal drug delivery system driven by TENG. Reproduced with permission.^[^ ^22^ ^]^ Copyright 2019, Elsevier.

Recent efforts in biomechanical energy research (see **Figure** **6**a)^[^ ^136^ ^]^ have utilized piezoelectric and triboelectric effects and applied them to wearable devices. When piezoelectric materials are squeezed or deformed during human movement, the material becomes polarized and generates a piezoelectric potential that produces circuit currents.^[^ ^137^ ^]^ The TENG was first invented to collect mechanical energy from the coupling of the triboelectric eﬀect and electrostatic induction.^[^ ^138^ ^]^ There are four basic modes of TENG that expand potential applications: lateral sliding mode, vertical contact‐separation mode, single‐electrode mode, and freestanding triboelectric‐layer mode.^[^ ^139^ ^]^ Recently, wearable TENGs have harvested biomechanical energy (from walking, running, jumping, bending, breathing, eye blinking, and even pulse waves) to drive self‐powered and mobile electronic devices (Figure 6b).^[140c]^

A self‐powered controllable transdermal drug delivery system based on a piezoelectric nanogenerator (PENG) can control drug release by collecting and converting mechanical energy into electrical energy. The delivery system can subcutaneously release 8.5 ng of dexamethasone with each electrical stimulation.^[^ ^141^ ^]^ A direct‐current fabric TENG with the most common plain structure is designed to harvest biomotion energy; a 1.5 cm × 3.5 cm TENG can quickly illuminate 416 serially connected LEDs.^[^ ^142^ ^]^ To effectively harvest low‐frequency biomechanical body energy, a linear‐to‐rotary hybrid nanogenerator was designed using a frequency enhancement strategy.^[^ ^143^ ^]^ The nanogenerator produced current and voltage that were enhanced up to 3.1‐fold and 3.6‐fold, respectively, at the base frequency (2 Hz).

Wearable TENGs are often deployed on feet or shoes to harvest the substantial kinetic energy and power output of high‐impact human movements.[^140^, ^144^] A self‐charging power unit based on cut paper can harvest and store body movement energy by integrating TENG technology and a supercapacitor.^[^ ^145^ ^]^ The energy can power wearable and portable electronic devices such as wireless remote controls, watches, or temperature sensors. To simultaneously harvest multiple forms of bioenergy, TENGs and glucose fuel cells have been hybridized to produce devices that improve electrical output and broaden energy sources.^[^ ^146^ ^]^ Because the TENG is relatively susceptible to ambient humidity, humidity‐resistant TENGs have also been developed.^[^ ^147^ ^]^ A high‐performance TENG that adapts to the environmental humidity from perspiration was fabricated with a unique electrostatic spinning nanofiber film; this TENG can produce an output current of 28 μA and a voltage of 345 V at 55% relative humidity.^[148c]^ A battery‐like self‐charging universal module consists of a power management unit and an energy collection unit and provides an excellent normalized output power of 2 mW g^−1^ at low frequencies (5 Hz).^[^ ^149^ ^]^

Beyond powering the sensors, bioenergy can also contribute directly to the therapeutic treatment process. For example, a therapeutic far‐red light source can be powered by the biomechanical energy harvested from a flexible implantable piezoelectric nanogenerator, and a self‐powered electro‐optogenetics system can effectively control glucose homeostasis.^[^ ^150^ ^]^ Mechanical stimulation is a ubiquitous method to trigger drug release from a carrier,^[^ ^148^ ^]^ and this stimulation can be powered by harnessed bioenergy.^[^ ^151^ ^]^ A touch‐driven transdermal drug delivery patch comprising a strain sensor and a refillable drug chamber with a microneedle array provides quantitative penetration in proportion to the extent of mechanical extrusion (Figure 6c).^[152b]^ A TENG‐based self‐powered implantable drug delivery system can provide in vitro *trans*‐scleral drug release in pig eyes.^[^ ^153^ ^]^ A pure hydrogel bioelectronic cardiac patch shows potential for implantable medical applications.^[^ ^154^ ^]^ Skin wound repair can be severely affected by bacterial infection, and wearable TENGs provide an interesting solution for healing infected skin wounds. A flexible TENG patch with surface‐engineered electrodes can accelerate infected wound healing via controlled drug release and local electrical stimulation (Figure 6d).^[^ ^155^ ^]^ Other TENG‐based devices trigger controlled drug release (doses up to 3 μg cm^−2^) when activated at 30–40 rpm for 1.5 min (Figure 6e)^[^ ^22^ ^]^ and can use electrical stimulation to regulate neural stem cell growth and differentiation.^[^ ^9^, ^13^ ^]^ A shape‐memory PENG can promote osteogenic differentiation and maintain bone homeostasis to treat osteoporosis and fractures.^[^ ^156^ ^]^ A biomechanical PENG‐powered photodynamic therapy system inhibits tumor growth via pulsed light stimulation.^[^ ^157^ ^]^

### 3.3 Biothermal Energy

**Figure 7:**
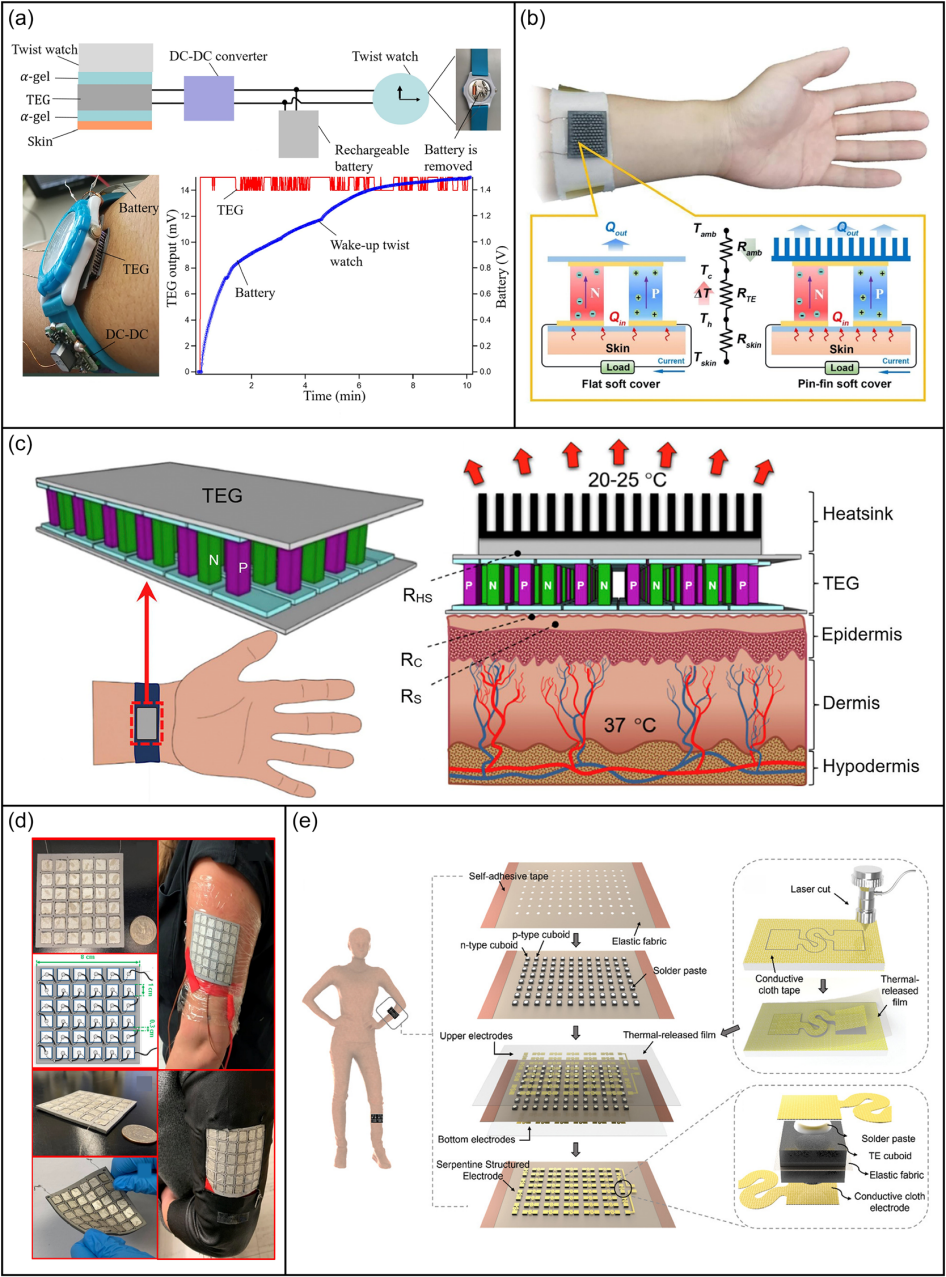
Biothermal energy in the closed‐loop medical system. a) Thermoelectric generator with a high integration density for portable and wearable self‐powered electronic devices. Reproduced with permission.^[^ ^159^ ^]^ Copyright 2021, Elsevier. b) Flexible wearable TED for human body thermal energy. Reproduced with permission.^[^ ^162^ ^]^ Copyright 2022, Elsevier. c) Wearable thermoelectric generator for human body thermal energy collection. Reproduced with permission.^[^ ^163^ ^]^ Copyright 2020, Elsevier. d) Wearable TEDs based on PEDOT:Tosylate/CuI paper composites. Reproduced with permission.^[^ ^164^ ^]^ Copyright 2021, American Chemical Society. e) Full‐fabric auxiliary TEDs for wearable electronic devices. Reproduced under the terms of the CC‐BY Creative Commons Attribution 4.0 International license (https://creativecommons.org/licenses/by/4.0).^[^ ^165^ ^]^ Copyright 2021, The Authors, published by Wiley‐VCH.

Much research has been devoted to powering wearable devices with energy harvested from the human body,^[^ ^15^, ^158^ ^]^ and human thermal energy can be directly converted into electricity by wearable thermoelectric devices (TEDs) that derive energy from ambient temperature fluctuations (**Figure** **7**a).^[^ ^159^ ^]^ Some TEDs can be attached to skin or embedded in clothing and these devices depend on temperature differentials with their surroundings to convert thermal energy into electrical energy.^[^ ^160^ ^]^ The produced thermoelectricity can power wearable sensors that detect finger movement and breathing patterns.^[^ ^161^ ^]^

A key challenge for wearable TEDs is the small temperature difference between the thermoelectric pillars, and conventional strategies use heat sinks and longer thermoelectric columns to increase the effective temperature differential. Such measures would reduce the comfort of wearable TEDs and hinder integration with clothing.^[^ ^162^ ^]^ To increase the output power of wearable TEDs, a pin‐fin soft cover with a large surface area can be used to reduce ambient‐side parasitic thermal resistance (Figure 7b).^[^ ^163^ ^]^

Wearable TEDs usually comprise p‐type and n‐type thermoelectric pillars connected in series and parallel. Common inorganic thermoelectric materials are rigid, bulky, and unsuitable for wearable devices. Recently, 2D thermoelectric materials with excellent performance have been developed, and a wearable TED with a fabric substrate can directly convert human thermal energy into electrical energy with an output power density of 6.63 μW cm^−2^ (Figure 7c).^[^ ^162^ ^]^ Wearable TEDs with a patterned serpentine‐shaped electrode on an elastic fabric substrate can tolerate strains up to 30%, thereby enabling high thermoelectric conversion efficiency with a thermal source of any shape. Using 36 pieces of a composite PEDOT:p‐toluene sulfonate/CuI paper (as P‐type element) and 36 wires (as N‐type element), a wearable TED can produce up to 4.7 μW of power at Δ*T* = 20 K and can be placed directly on the skin or clothing (Figure 7d).^[^ ^164^ ^]^ An ultraflexible fabric‐based thermoelectric generator with a conductive fabric electrode and an elastic fabric substrate provides excellent structural integrity and flexibility while harnessing biothermal energy at strains up to 30% and on arbitrarily shaped heat sources. This device generates power up to 64.10 μW and an output voltage of 111.49 mV with a temperature difference of 33 K (Figure 7e),^[^ ^165^ ^]^ and energy density can be increased by integrating many collecting modules together. Biothermal energy can facilitate drug delivery by combining a conductive hydrogel with a thermally responsive poly(*n*‐isopropylacrylamide) hydrogel to form a double layer that functions as two actuators because of the variable expansion and contraction introduced by varying thermal stimulation.^[^ ^76^ ^]^ Drug loading and release in poly(*n*‐isopropylacrylamide‐grafted acrylic acid) hydrogel‐functionalized textiles require the expansion and contraction of hydrogels. The concentration of hydrophilic copolymer acrylic acid in the hydrogel can be varied to enable drug storage at room temperature and on‐demand release with mild thermal stimulation.^[^ ^31^ ^]^

## 4 Bioenergy‐Based Closed‐Loop Medical System

Closed‐loop medical systems enable precision personalized medicine.^[^ ^166^ ^]^ Therapeutics are often administered as multiple treatments over a period of time in which patient physiology and disease pathology can change, and real‐time monitoring would enable treatment plan design and optimization. Increasingly advanced computer technologies are used to analyze physiologic sensor signals and provide more accurate feedback on treatment effects.^[^ ^18^ ^]^ Bioenergy‐based therapeutic sensing systems obviate the need for complex line connections and frequent power supply replacements, thus facilitating wearable device integration and personalized medicine.^[^ ^167^ ^]^ Wearable treatment systems will improve therapeutics, particularly for diseases of relatively fragile organs such as the eyes and ears.[^4^, ^16^, ^40^]

**Figure 8:**
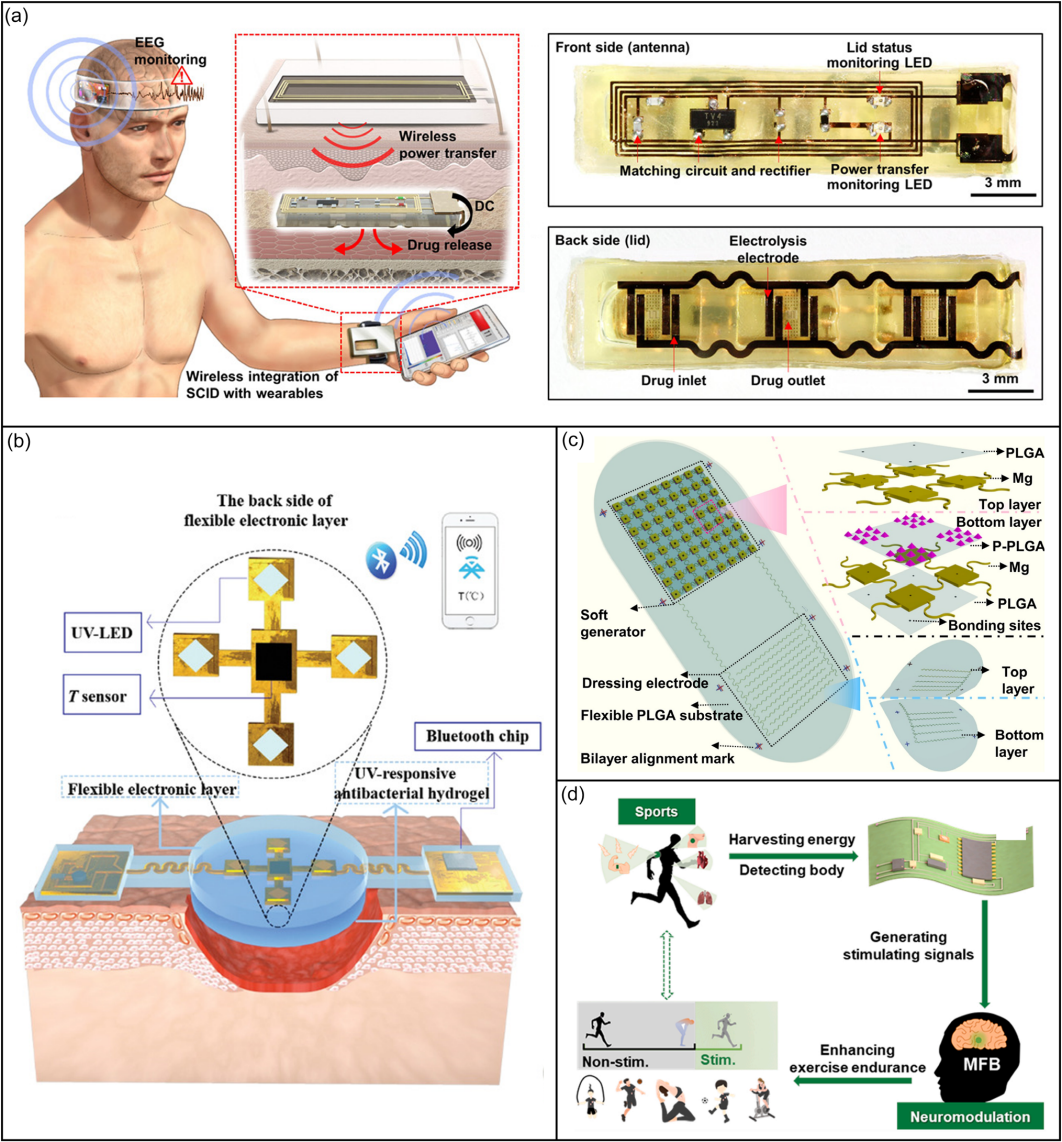
Application implementation modes of the closed‐loop medical system based on bioenergy. a) Soft implantable drug delivery device integrated wirelessly with wearable devices to treat fatal seizures. Reproduced with permission.^[^ ^170^ ^]^ Copyright 2021, The Authors, published by AAAS. b) Smart flexible electronics‐integrated wound dressing for real‐time monitoring and on‐demand treatment of infected wounds. Reproduced under the terms of the CC‐BY Creative Commons Attribution 4.0 International license (https://creativecommons.org/licenses/by/4.0).^[^ ^30^ ^]^ Copyright 2020, The Authors, published by Wiley‐VCH. c) Self‐powered implantable bioabsorbable electrical stimulation device for fracture healing. Reproduced with permission.^[^ ^171^ ^]^ Copyright 2021, The Authors, published by National Academy of Sciences, USA. d) Self‐powered wearable system improves athletic endurance performance. Reproduced with permission.^[^ ^120^ ^]^ Copyright 2022, Elsevier.

Smart wound dressings with integrated near‐field communication modules can harvest energy and transmit data wirelessly to enable on‐site signal processing and drug delivery control via smartphones.^[^ ^168^ ^]^ Such sensors can assess wound condition by simultaneously measuring wound temperature, pH, and uric acid concentration, while electrodes in the dressing deliver antibiotics on‐demand using electrical control.^[^ ^30^ ^]^ Multifunctional hydrogel composites of polyacrylamide, quaternary ammonium chitosan, carbon quantum dots, and phenol red enable highly responsive, reversible, and accurate colorimetric pH sensing that allows real‐time UV and visible light imaging of wound pH and dynamics.^[^ ^169^ ^]^ A unified drug delivery system based on the Medical Internet of Things can automatically detect and control seizures using a cointegrated epilepsy detection unit and a drug delivery unit. Seizure detection is performed in real time using statistical feature extraction and deep neural network classifiers, after which the drug is delivered by a piezoelectric‐driven valveless dual‐reservoir micropump.^[^ ^6^ ^]^ A soft implantable drug delivery device can be integrated wirelessly with a wearable sensor to monitor electroencephalography signals and trigger subcutaneous drug release via wireless voltage induction (**Figure** **8**a).^[^ ^170^ ^]^ An intelligent and flexible wound dressing with electronics and sensors integrated within a double‐layer structure can provide real‐time wound temperature monitoring and early infection diagnosis and deliver antibiotics on demand via in situ UV irradiation of the hydrogel (Figure 8b).^[^ ^30^ ^]^

Shoe insoles can be integrated with electronic textile‐based pressure sensors and low‐cost inertial measurement units (comprising a three‐axis accelerometer, gyroscope, and magnetometer) to measure plantar pressure and gait characteristics, respectively. A smartphone interface receives and displays the Bluetooth‐transmitted real‐time sensor data for use in healthcare, rehabilitation, therapy, and sports training.^[^ ^83^ ^]^ A self‐powered, implantable, and bioabsorbable electrostimulation device enables closed‐loop biofeedback‐based bone fracture healing; the device uses a TENG for electricity generation and a pair of dressing electrodes to apply electrical stimulation to the fracture (Figure 8c).^[^ ^171^ ^]^ Tissue and organ movement near the dressing provides bidirectional electrical pulses that activate growth factors and promote bone regeneration. Wearable systems can monitor real‐time vital signs and transmit neurostimulation signals to the brain. Piezoelectric generators can harvest the kinetic energy of athletes by converting mechanical energy into electricity. A device made with flexible piezoelectric polymers can monitor respiration, the carotid artery, and heartbeat and process these physiologic data to trigger neural stimulation.^[^ ^116^ ^]^ Such a device can enhance endurance performance in running mice when the stimulating electrodes are implanted in the medial forebrain bundle (Figure 8d).^[^ ^120^ ^]^ An ultrathin, flexible, and comfortable sweat‐activated battery with high power density (16.3 mW cm^−2^) and energy capacity (74.4 mAh) can illuminate 120 LED lights continuously for over 4 h. The device also provides enough power to support wireless physiologic sensing for over 1.2 h.^[140f]^ Wearable heart rate detectors can monitor real‐time heart health and wirelessly transmit the data to medical personnel for analysis and feedback. A flexible, comfortable, and breathable electronic skin can be used for long‐term daily physiological signal monitoring and diagnosis; this smart wearable ECG system is a “portable doctor” that monitors health at any place and time.^[^ ^91^ ^]^

Ongoing developments in bioenergy‐based closed‐loop systems will revolutionize medical treatment with flexible and wearable smart devices that were previously possible only in science fiction stories. Personal lives will gradually be filled with masks that analyze emotional value, contact lenses that relieve intraocular pressure and treat eye disease, smart bandaids that release medicines on demand, and other innovations. Each person can become a “personal doctor” that uses their smartphone to obtain health and disease information in real time.

## 5 Conclusion

Closed‐loop bioenergy‐based medical devices are being actively developed for their immense potential in modern clinical medicine and human health monitoring. Although impressive progress has been made, significant challenges remain in developing devices with rapid, high‐resolution, high‐sensitivity monitoring, and fast‐response therapy. These challenges arise from the difficulty of integrating a stable energy supply, wireless sensing, and drug delivery modules on a flexible device made of biocompatible materials. Wearable medical devices must fit properly with the human body for extended periods during complex movements to collect accurate information and harness bioenergy. In extreme environmental conditions such as high humidity, the output stability of the device requires further improvement. The detection accuracy of wearable devices is critical for serious and complex diseases. Despite these challenges, closed‐loop medical systems have already demonstrated remarkable potential for convenient and effective medical treatment and inspired researchers toward further in‐depth studies and breakthroughs. This research field will require a complex multidisciplinary approach that incorporates advances in machine learning, wireless sensing, and drug research and development, and artificial intelligence will be particularly critical for the analysis and processing of sensor data. We believe these developments in closed‐loop medical systems will create many opportunities to realize the potential for a more intelligent, precise, and personalized medical system.

## Conflict of Interest

The authors declare no conflict of interest.
